# Metabolic engineering of hairy root cultures in *Beta vulgaris* for enhanced production of vanillin, 4-hydroxybenzoic acid, and vanillyl alcohol

**DOI:** 10.3389/fbioe.2024.1435190

**Published:** 2024-10-02

**Authors:** Zakir Husain, Zafar Iqbal Warsi, Sana Khan, Ganesan Mahendran, Shama Afroz, Ashish Chandran, Praveen Kumar Kashyap, Kahkashan Khatoon, Gazala Parween, Sudeep Tandon, Laiq Ur Rahman

**Affiliations:** ^1^ CSIR-Central Institute of Medicinal and Aromatic Plants (CIMAP), Lucknow, Uttar Pradesh, India; ^2^ Phytochemistry Division, CSIR-Central Institute of Medicinal and Aromatic Plants, Lucknow, Uttar Pradesh, India

**Keywords:** VpVAN, hairy root, elication, vanillin, vanillyl alcohol, 4-Hydrobenzoic acid

## Abstract

The flavor of vanilla is a complex blend of compounds, with vanillin as the most prominent, along with vanillyl alcohol and 4-hydroxybenzoic acid. Natural vanillin extracted from vanilla beans is expensive, so researchers use heterologous synthesis to produce nature-identical vanillin in plant hosts. Consequently, alternative traditional farming and gathering methods are required to bridge the significant disparity between supply and demand. The current research successfully developed a method to induce hairy root formation from leaves. It integrated the Vanillin synthase (VpVAN) gene into transgenic hairy root lines of *Beta vulgaris*, synthesizing vanillin-related compounds. The presence of the VpVAN gene in transgenic roots was confirmed using PCR analysis. Additionally, RT-qPCR analysis demonstrated the expression of the VpVAN gene in the transgenic root lines. The transgenic hairy root clones H1, H2, and H5 showed enhanced vanillin production, vanillyl alcohol, and 4-hydroxybenzoic acid. Elicitation with methyl jasmonate (MJ) and salicylic acid (SA) further improved the production of these compounds in *B. vulgaris* hairy roots. The maximum hairy root biomass was observed after 60 days, with the maximum synthesis of vanillin and 4-hydroxybenzoic acid obtained from hairy root clones H5 and HR2, respectively. Vanillyl alcohol HR2 was obtained on the 45th day of cultivation. Elicitation with wound-associated hormone methyl jasmonate and salicylic acid enhanced the yield of vanillin, vanillyl alcohol, and 4-hydroxybenzoic acid, with a 215-fold increase in vanillin, a 13-fold increase in vanillyl alcohol, and a 21 fold increase in 4-hydroxybenzoic acid. The study results indicate that establishing transgenic hairy root cultures with the VpVAN gene is a promising alternative method for enhancing the production of vanilla flavor compounds such as vanillin, vanillyl alcohol, and 4-hydroxybenzoic acid. A cost-effective protocol has been developed to mass-produce phenolic compounds using a hairy root culture of *B. vulgaris*. This approach addresses the increasing demand for these substances while reducing the cost of natural vanillin production, making it suitable for industrial-scale applications.

## 1 Introduction

Vanillin, also known as 4-hydroxy-3-methoxybenzaldehyde, is the principal constituent of vanilla bean extract, derived from the pods of various species of vanilla orchids, primarily *Vanilla planifolia*, its sweet and creamy aroma is highly prized, making it a defining characteristic of vanilla flavor, natural vanillin, however, represents less than 1% of global production due to the labor-intensive and expensive extraction process (Gallage et al., 2014). The vanilla flavor is composed of a complex mixture of compounds; compounds in higher concentrations include vanillin, vanillic acid, vanillyl alcohol, p-hydroxybenzoic acid, carbohydrates, lipids, and other substances (Sinha et al., 2008) from *V. planifolia*. However, the majority are produced chemically from fossil fuels or through lignin hydrolysis in acid (Zamzuri and Abd-Aziz, 2013; Singh et al., 2015). Therefore, there is a need to develop new methods for producing naturally sourced vanillin that meet these requirements. According to EU and US law, vanillin derived from genetically modified can be labeled natural vanillin (Waltz, 2015). The approach involves the biotransformation of plant-derived materials using plant cells or microbial systems’ enzymatic machinery to produce vanillin (Madhavan et al., 2019; Kallscheuer et al., 2019). The alternative method involves using metabolic engineering to incorporate genes from the phenylpropanoid pathway in either plants or microbes, this allows for vanillin production from precursor molecules, including intermediate stages (Arya et al., 2022; Chee et al., 2017), most biotechnological processes used to produce vanillin depend on microorganisms such as yeast, fungus, and bacteria, which act as production hosts to bioconvert natural substances like lignin, ferulic acid, eugenol, and isoeugenol, among others (Kaur and Chakraborty, 2013). Vanillyl alcohol or 4-hydroxy-3-methoxybenzyl alcohol, is phenolic alcohol obtained from vanillin and occurs naturally (Lee et al., 2023); its applications include its use as a flavoring agent in food and beverages due to its pleasant vanilla-like aroma, as well as in perfumes and cosmetics (Ravier et al., 2024), vanillyl alcohol has antioxidant and antimicrobial properties, making it valuable in pharmaceutical formulations (Zieniuk et al., 2022). Industrially, it is an intermediate in producing various other chemicals and compounds (Priefert et al., 2001). The market value of vanillyl alcohol is influenced by factors such as demand in the flavor and fragrance industry, natural source availability, and advancements in synthetic production methods (Khoyratty et al., 2018).

4-Hydroxybenzoic acid (4-HBA) holds significant importance across various industries, serving as a vital precursor to parabens, a key component in drug synthesis, and a fundamental building block in producing specialty polymers and resins (Braga and Faria, 2020). Its versatile applications, especially in the cosmetics and pharmaceutical sectors, drive its market value (Yu et al., 2016). Within plant biology, 4-HBA plays a crucial role in secondary metabolite production, displays antioxidant properties, and contributes to structural integrity (Parvin et al., 2022). Moreover, it actively participates in plant defense mechanisms, growth regulation, and allelopathy (Wei et al., 2023).

Previous studies suggest that biotic and abiotic factors can stimulate a plant response, enhance bioactive compound production, and encourage growth in plant cells, tissues, and organs (Thakur et al., 2019; Mahendran et al., 2022). Elicitors, functioning as external defense proponents, initiate a cascade of physiological and biochemical reactions that activate intracellular signal transduction pathways, these pathways subsequently lead to the production of bioactive metabolites (Gai et al., 2019; Guru et al., 2022; Jha and Mohamed, 2022). Recent studies suggest that utilizing certain substances, such as salicylic acid, methyl jasmonate/jasmonic acid, silver nitrate, cadmium chloride, yeast extract, and chitosan, could enhance the production of secondary metabolites in hairy root culture (Naik and Al-Khayri, 2016; Hesamiet al., 2023). A practical method for increasing vanillin content in Capsicum frutescens callus cultures is through the heterologous expression of a *VpVAN* gene. The transformed tissues showed a vanillin content of 0.057%, significantly higher than the 0.0003% in untransformed tissues (Chee et al., 2017). Furthermore, transgenic rice cell cultures produced varying amounts of vanillin; the highest yield was 544.72 μg/g of fresh calli (Arya et al., 2022). This information highlights the potential of biotechnological methods in producing vanillin and their practical applications. This study explores a possible plant-based solution for producing vanillin and related compounds like Vanillyl alcohol and 4-Hydroxybenzoic acid. The approach involves expressing the VpVAN gene in hairy roots of *B. vulgaris* and conducting comparative research; ferulic acid could serve as a precursor for the biosynthesis of vanillin and other phenolic compounds; thus, it may be possible to achieve higher conversion rates from endogenous ferulic acid to vanillin and related compounds vanillyl alcohol and 4-hydroxybenzoic acid in *B. vulgaris* by constitutively expressing VpVAN, compared to untransformed plants. The research shows that bioengineered plant-based systems can provide natural vanillin, vanillyl alcohol, and 4-hydroxybenzoic from different food crops.

## 2 Materials and methods

### 2.1 Plant material

The *B. vulgaris* seeds were thoroughly washed with 70% ethanol and then surface-sterilized using a solution containing 0.1% HgCl_2_ for 4 min. After treatment, the seeds were carefully washed with autoclaved water before being placed on a basal MS medium supplemented with 3% sucrose and 0.6% agar. Therefore, following seed germination and once the plants reached a sufficient height for leaf development, the leaves were used as the primary material for subsequent experiments focusing on leaf-specific hairy root formation.

### 2.2 Genetic constructs for vanillin and analog production

The mRNA sequence for vanillin synthase (VpVAN; GenBank: KP278240.1) in *V. planifolia* was retrieved from the GenBank database (NCBI, United States). Total RNA was extracted from a *V. planifolia* pod using a qiagen total RNA Kit, and cDNA was synthesized utilizing a cDNA synthesis kit (Pure Gen). The cDNA served as a template for amplifying the VpVAN gene with gene-specific primers (F: 5′-ATG​GAT​CCC​AAA​TTG​CTA​TTC​C-3′) and (R: 5′- GAG​AGT​CGC​AGG​GTT​AGG​TCT​G -3′), resulting in a 1,071 base pair amplicon. The amplified DNA fragment was then cloned into a pGEM-T Easy vector and sequenced for verification. Sequence alignment between the VpVAN and cloned sequences was performed using Clustal-w. Subsequently, the pGEM-T Easy vector carrying the VpVAN gene was digested with XbaI and BamHI, and the VpVAN fragment was purified using a gel extraction kit (GeNeiTM™). The verified amplicon was cloned into the binary vector pBI121 at the XbaI/BamHI site under the regulation of the CaMV 35S promoter. The recombinant plasmid, pBI121-VpVAN, was successfully introduced into *Escherichia coli* (DH5α strain) and *Agrobacterium rhizogenes* 9,402 using the freeze-thaw transformation method. Colony PCR with gene-specific primers confirmed the presence of the pBI121-VpVAN constructs in *A. rhizogenes* cells following transformation. This genetic construct is used to induce hairy root formation in *B. vulgaris* *.*

### 2.3 Standardize the genetic transformation of *Beta vulgaris*

Before proceeding to develop transgenic hairy roots in *B. vulgaris*, optimizing various factors that influence *A. rhizogene*-mediated genetic transformation is important, and these factors include determining the optimum kanamycin concentration for selecting putative transformants, the optimal bacterial optical density, finding the ideal acetosyringone concentration, and identifying the best co-cultivation time. Thorough optimization of these parameters is necessary to ensure the efficiency and success of the transformation process.

#### 2.3.1 Optimization the effects of *Agrobacterium rhizogenes* cell densities on hairy root induction

The root transformation experiments implicated using the LBA 9402 strain of *A. rhizogenes*. A medium containing yeast extract and mannitol was used to cultivate *A. rhizogenes*; it included 1.0 g/L yeast extract, 0.5 g/L dipotassium phosphate, 0.2 g/L magnesium sulfate, and 0.1 g/L sodium chloride, with agar at a concentration of 15.0 g/L. To begin thawing the cryopreserved glycerol stock of *A. rhizogenes* LBA 9402, it was streaked onto agar plates containing YEM medium and subsequently incubated at 28°C for 48 h. To begin thawing the cryopreserved glycerol stock of *A. rhizogenes* LBA 9402, it was streaked onto agar plates containing YEM medium and subsequently incubated at 28°C for 48 h. A bacterial colony was selected and cultured in 50 mL of YEM medium. It was then incubated at 180 rpm and 28°C for 16–24 h. The bacterial culture’s optical density (OD) was measured to ensure it reached the optimal range of 0.6–0.8; once it reached this density, it was harvested via centrifugation at 4,000 rpm for 10 min. The supernatant was discarded, and the cell pellets were washed and resuspended in a half-strength liquid MS basal medium.

#### 2.3.2 The optimization of bacterial cell density, infection duration, and acetosyringone concentration

Leaves obtained from the *in vitro* culture of *B. vulgaris* are used as an explant for hairy root initiation. The leaves are placed on the abaxial side in a Petri dish and then punctured with a 1 mL syringe needle loaded with a suspension of *A. rhizogenes* cells, specifically the LBA9402 strain. To ensure uniform bacterial cell density, the *A. rhizogenes* cell suspension is applied to the leaves for 15 min at five different optical densities, measured at OD_600_ nm (0.2, 0.4, 0.6, 0.8, or 1.0). The study also examines the effect of the duration of infection time on hairy root induction. The leaves are injured and exposed to the *A. rhizogenes* cell suspension (cell density of OD_600_ = 0.8) for varying durations, including 5, 10, 15, 20, and 25 min. Investigation is carried out to determine the impact of varying concentrations of acetosyringone (50, 100, 150, 200, and 250 µM) added to the *A. rhizogenes* cell suspension on the development of hairy roots.

#### 2.3.3 Establishment of transgenic hairy root

After infecting the leaves with *A. rhizogenes*, the excess suspension was removed by drying them on filter paper. The infected leaves were then transferred to MS medium. After 48 h, the infected leaves were moved to an MS medium containing 250 mg/L cefotaxime and 15 mg/L kanamycin to prevent the growth of *A. rhizogenes*.

### 2.4 Hairy root induction

The *pBI121*-*VpVAN* plasmid was cultured in Yeast Mannitol Broth with 25 mg/L kanamycin, using *A. rhizogenes* strain (9,402) at 28°C and 180 rpm overnight. The bacterial culture was centrifuged at 10°C for 7 min at 4,000 rpm. After centrifugation, the culture was resuspended in a liquid MS medium to inoculate the explants with the bacterial culture, and a needle was used to pierce the leaves to initiate hairy root formation from the wound site. The leaves were carefully dried using a sterile filter paper and kept in a dark environment at a temperature of 25°C ± 2°C on a semisolid, hormone-free MS medium. After 48 h of co-cultivation, the explants were shifted to an MS medium containing kanamycin 15 mg/L and 250 mg/L cefotaxime to eliminate excess bacterial growth. Two weeks after the injection, the wounded explant sites demonstrated the development of hairy roots. The hairy roots were then separated from the explant tissue and moved to a semisolid medium containing cefotaxime (250 mg/L) before being put in a rotary shaker. The fast-growing roots were then transferred to 250 mL flasks containing 50 mL of liquid medium with 30 g/L sucrose. The hairy root cultures were kept dark at 22°C ± 5°C and 90 rpm.

### 2.5 Molecular analysis of the hairy roots of *Beta vulgaris*

The PCR analysis confirmed the incorporation of the VpVAN and rolC genes into the transgenic hairy root lines. Total genomic DNA isolated from transgenic hairy root lines using the CTAB method. Then, we used specific primers targeting the respective genes to analyze potential transformant lines that successfully amplified the VpVAN gene using primers (Supplementary Table S1). To amplify the target sequence, the PCR protocol involved a primary denaturation step at 94°C for 4 min, followed by 30 cycles of secondary denaturation at 94°C for 30 s, annealing at 62°C for 30 s, and elongation at 72°C for 1 min and 30 s. A final extension step was performed at 72°C for 10 min. For the amplification of the rolC gene, a forward and reverse primer was used. The PCR protocol involved a primary denaturation step at 94°C for 5 min, followed by 30 cycles of secondary denaturation at 94°C for 30 s, annealing at 55°C for 30 s, and elongation at 72°C for 1 min. A final extension step was performed at 72°C for 10 min.

### 2.6 Real-time quantitative PCR for analyzing gene expression

The genomic RNA was isolated from the transformed hairy root lines and the non-transformed (wild-type) roots using the qiagen plant mini kit; for quantification using a nanodrop spectrophotometer, the RNA samples underwent cDNA synthesis using the Puregene cDNA synthesis kit. The Primer 3 software was used to design primers with a length of around 20 to 22 nucleotides, a melting temperature exceeding 50 °C, a GC content ranging from 40% to 60%, and an anticipated amplicon size between 90 and 120 base pairs. The primers were explicitly designed for real-time applications. After that, the concentration of cDNA was maintained within the range of 150–200 ng/μL precision; the gene-specific primers were used (Supplementary Table S1), and semi-quantitative PCR was performed to compare gene expression. The actin gene of B. vulgaris used to normalize the expression of the target gene was used to determine the relative fold by using 2−^ΔΔCt^ (Afroz et al., 2022, 2023; Warsi et al., 2023). To ensure the credibility of the data, all reactions were repeated three times.

### 2.7 Growth kinetics of hairy roots

Four distinct hairy root lines were observed to determine their growth duration to assess their growth kinetics, 5-7 roots, and each measuring 2 cm in length and weighing between 0.5 and 0.6 g; these roots were then introduced into 50 mL of liquid MS medium, and the media were incubated on a shaker at 90 rpm. The hairy roots fresh weight (FW) and dry weight (DW) were measured at various time intervals of 15, 30, 45, 60, and 75 days. The hairy root lines were dried using filter paper to remove excess water. The fresh weight of the hairy root lines was analyzed, and after drying them in an oven at 50°C for 24 h, their dry weight was determined.

### 2.8 Effect of MJ, SA, on the accumulation of increasing vanillin, vanillyl alcohol, and *4*-hydroxybenzoic acid

After 60 days of old hairy root cultures reaching the stationary phase stage, an elicitation experiment was conducted to determine the impact of elicitors on enhancing the levels of vanillin, vanillyl alcohol, and p-hydroxybenzoic acid. The experiment involved adding varying concentrations of methyl jasmonate (50, 100, 150, and 200 μM) and Salicylic acid (50, 100, 150, and 200 μM), and the efficacy of each concentration was evaluated. A control group of hairy root cultures without an elicitor was also used. The levels of vanillin, vanillyl alcohol, and p-hydroxybenzoic acid were measured after 48 h for each elicitor concentration.

### 2.9 Extraction procedure for HPLC analysis of root samples

To perform the extraction, 100 mg of control and transgenic (air-dried) were taken from both *in vitro* (7-9-week-old) grown hairy roots. These samples were subjected to quantitative phenolic, dried roots were ground into a powder using a mortar and pestle. Then, 0.1 g of the powder was extracted with methanol (3 × 10 mL) and placed in an ultrasonic bath at 25°C for 25 min, followed by centrifugation at 11,000 rpm for 15 min. The supernatant was vacuum-dried and redissolved in 1.0 mL of methanol. The redissolved extract was centrifuged (11,000 rpm for 15 min) and passed through a syringe filter; the filtrate was used for further experiments.

### 2.10 Levels of phenolic compounds detected in the hairy roots of *Beta vulgaris*

The root extracts for HPLC analysis were prepared following established protocols. The extracts were dissolved in 1 mL of HPLC-grade methanol and passed through a 0.22 µm syringe filter. These samples were then analyzed for vanillin, vanillyl alcohol, and p-hydroxybenzaldehyde using a Waters modular HPLC system. The system comprised a 2,996 photodiode array detector, a 600 E pump with manual injection, and a Waters C18 XBridge column. A solvent system of solvent A (water with 0.1% acetic acid) and solvent B (99.9% acetonitrile) was used for gradient elution at a flow rate of 0.8 mL/min during a 40-min analysis period. The gradient program started with 90% A and transitioned to 77% a over 30 min, maintained for the remaining 5 min. All chemicals were sourced from sigma–aldrich. The data presented represent the average of three independent experiments conducted in triplicate.

### 2.11 Statistical analysis

The study on hairy root cultures was carried out in triplicate, and each experiment reported the average values and standard deviation of three replicates. The quantification was performed twice with three replicates. Tukey’s HSD test was conducted using IBM SPSS statistics software (version 22.0, United States) to establish the statistical significance of the differences in the results.

## 3 Results and discussion

### 3.1 The impact of different cell densities of *Agrobacterium rhizogenes* on the induction of hairy roots

**FIGURE 1:**
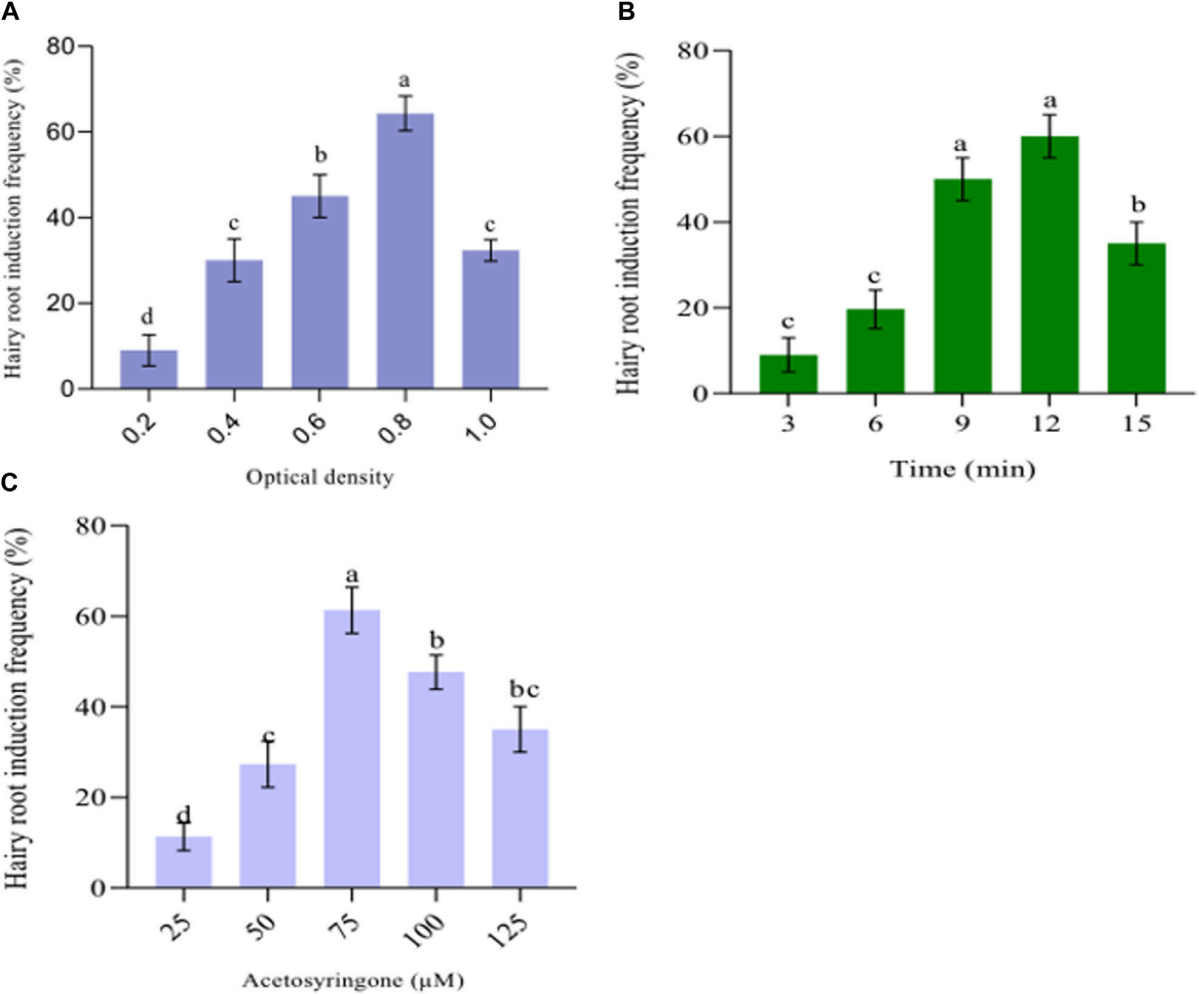
Factors affecting hairy root induction include cell density, infection duration, and acetosyringone concentration. **(A)** The optical cell density of *Agrobacterium rhizogenes*. **(B)** *Agrobacterium rhizogenes* infection time. **(C)** Acetosyringone concentration’s effect on hairy root induction. The data provided the mean ± standard deviation. Suppose a letter is used to represent values in a particular experiment. The same letter in an individual experiment indicates no significant difference by Tukey’s test (p < 0.05).

**FIGURE 2:**
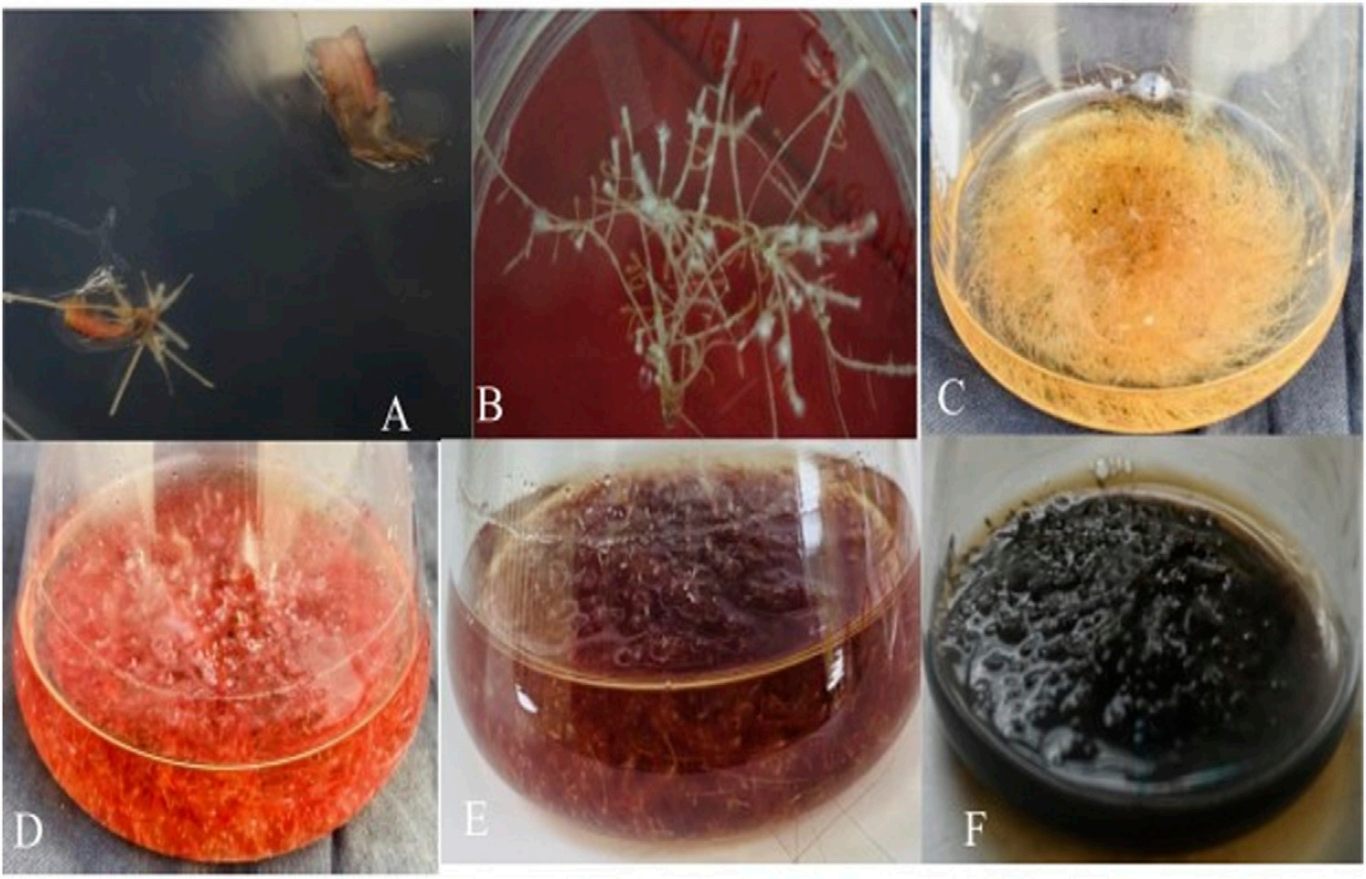
Induction and establishment of *Agrobacterium rhizogenes* strain (LBA 9402) mediated hairy root culture from *Beta vulgaris* leaf explant. **(A)** Hairy root induction from infected site of leaf explants **(B)** The thin and branched hairy root line. **(C)** 30 days old hairy root culture. **(D)** 45 days old hairy root culture. **(E)** 60 days old hairy root culture. **(F)** 75 days old hairy root culture.

Series of experiments were conducted to determine the optimal density of *A. rhizogenes* suspension for inducing root formation. The results (Figure 1A) demonstrate that the highest efficiency of root induction, with 64.33% ± 4.041% success rate, was achieved when using a density of 0.8 (OD_600_) (Figure 2A). When the cell density was increased to 1.0, the efficiency of root induction decreased from 64.33% ± 4.041% to 32.33% ± 2.157% (Figure 1A). Our research discovered that exposing leaf explants to insufficient bacterial cell density (0.2 at OD_600_) resulted in a transformation frequency of at least 9.00% ± 3.60%. Conversely, higher cell densities (1.0 at OD_600_) increased T-DNA attachment from bacteria to host cells, causing the explants to turn brown and reducing the frequency of hairy root induction. Similarly, the optimal hairy root induction efficiency occurred at a cell density of 0.6–1.0 (OD_600_) in the suspension of *A. rhizogenes* with an OD_600_ of 0.8; this suspension was utilized for infection in L. chinensis cv. “Fenhongguiwei” (Qin et al., 2021). Other studies have reported similar findings with different plant species, including Solanum trilobatum (OD_600_ = 1.0) (Shilpha et al., 2015), Rauwolfia serpentine (OD_600_ = 0.6) (Bhagat et al., 2019), Bacopa monnieri (OD_600_ = 0.6) (Bansal et al., 2014), Ocimum sanctum (OD_600_ = 0.8) (Sharan et al., 2019), common bean (Phaseolus vulgaris L.) (OD_600_ = 0.5) (De Koning et al., 2023). Developing plant roots, enhancing biomass and biosynthesizing secondary metabolites can be achieved using successful techniques like hairy root induction and *A. rhizogenes*-mediated transformation (Rawat et al., 2019). The process involves infecting *A. rhizogenes* to induce hairy roots. The transformation of rol genes depends on vir gene activation and insertion of T-DNA genes into the host cells. To optimize hairy root induction, it is essential to infect the appropriate *A. rhizogenes* strain for host specificity (Sathasivam et al., 2022). The density of cells influences the effectiveness of *A. rhizogenes* in inducing root growth (Moehninsi and Navarre, 2018; Mahendran et al., 2022).

### 3.2 Impact of infection duration on the formation of hairy roots

The frequency of root induction may vary depending on the time of infection with *A. rhizogenes*. Studies suggest that the optimal timing for root induction varies with the bacterial strain and plant species (Ooi et al., 2013). In our study, after conducting tests on four different infection times, we found that the 12-min infection time was the most suitable for inducing root growth, with an average rate (60.00 ± 5.07) (Figure 1B). The findings of the current study suggest that explants infected for a brief period (3 min) had a lower frequency of root induction compared to those infected for a longer duration and on the other hand, infection time beyond 15 min reduced root induction frequency due to excessive growth of *A. rhizogenes* on the surface of the leaf explant, causing necrosis and inhibiting root induction. Researchers have reported comparable findings where 5 min was effective for Allium sativum, (Phuong et al., 2023), and 10 min for Lythrum salicaria (Ebrahimi et al., 2023), 15 min for Perilla frutescens (Yan et al., 2023) 20 min for Curcuma longa (Sandhya and Giri, 2022), and 30 min for Semecarpus anacardium (Panda et al., 2017).

### 3.3 Acetosyringone concentrations affect root induction

In order to increase the likelihood of hairy root formation, we applied varying concentrations (25, 50, 75, 100, and 125 µM) of acetosyringone, a compound that activates vir genes, prior to infection. The results of the experiment showed that the leaf explants treated with 50 µM of acetosyringone had the hair root induction efficiency (40.33% ± 3.64%), (Figure 1C). Conversely, the infecting medium containing 75 µM of acetosyringone yielded the highest root induction efficiency (61.33% ± 5.132%). This outcome has been reported before (Mohiuddin et al., 2009; Mahendran et al., 2022). To enhance the efficiency of hairy root induction, a concentration of 75 µM acetosyringone was added during either the infection time or co-cultivation with *A. rhizogenes*. Similar findings with different plant species, including *Plumbago auriculata* (acetosyringone 100 µM) (Zhao et al., 2023), *Rubi*a *yunnanensis* (acetosyringone 50 µM) (Miao et al., 2021).

### 3.4 Molecular analysis of the hairy roots of *B. vulgaris*

**FIGURE 3:**
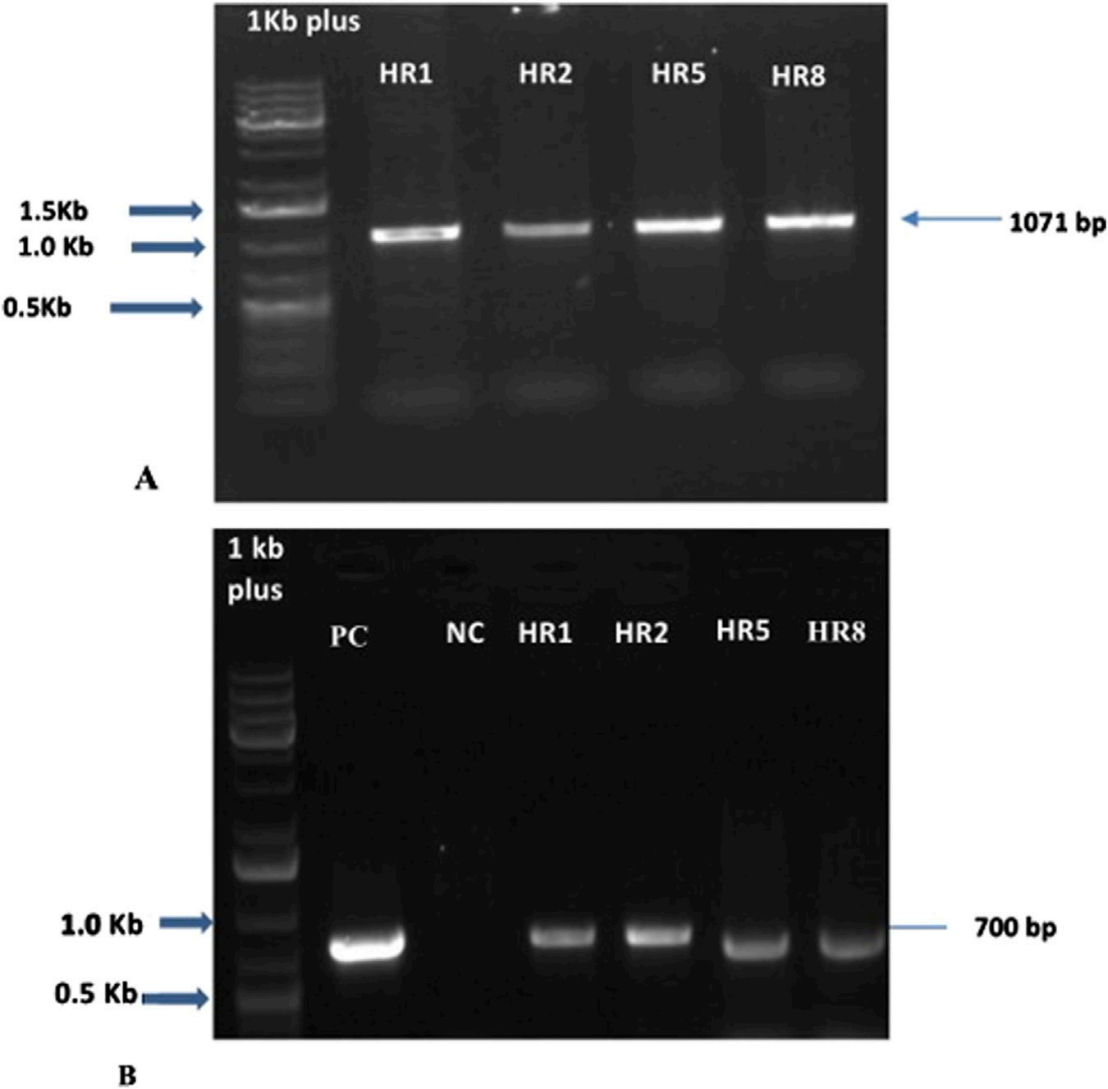
The putative transformants’ hairy root lines. **(A)** PCR amplification of the VpVAN gene produced 1071 bp of the amplicon. M- Marker (1 kb plus ladder), HR1, HR2, HR5, and HR8 are the putative transformants. **(B)** PCR amplification of the rol C gene produced 700 bp of the amplicon, PC - Positive Control, NC -Negative Control, HR2, HR3, HR5, and HR8.

In this study, we have validated the integration of root growth-stimulating genes, namely root-inducing T-DNA genes and VpVAN gene, into the transformed hairy root line DNA using the rolC gene and VpVAN gene. We observed a 1,072 bp for the VpVAN gene and size of 700 bp for rolC in the generated hairy root lines (Figures 3A, B). Previous research on *B. vulgaris* (Rahman et al., 2009; Singh et al., 2015) has also reported the presence of rolC gene in hairy root lines. The rol genes, such as rolA, rolB, rolC, and rolD, have a significant role in hairy root induction, root development, and phenotype line formation (Nilsson and Olsson, 1997; Aoki and Syōno, 2000; Casanova et al., 2005). The specific proteins in rol genes trigger the synthesis of cytokinin and auxin in the converted plant or roots (Bulgakov et al., 2018).

### 3.5 Gene expression analysis through real-time PCR

**FIGURE 4:**
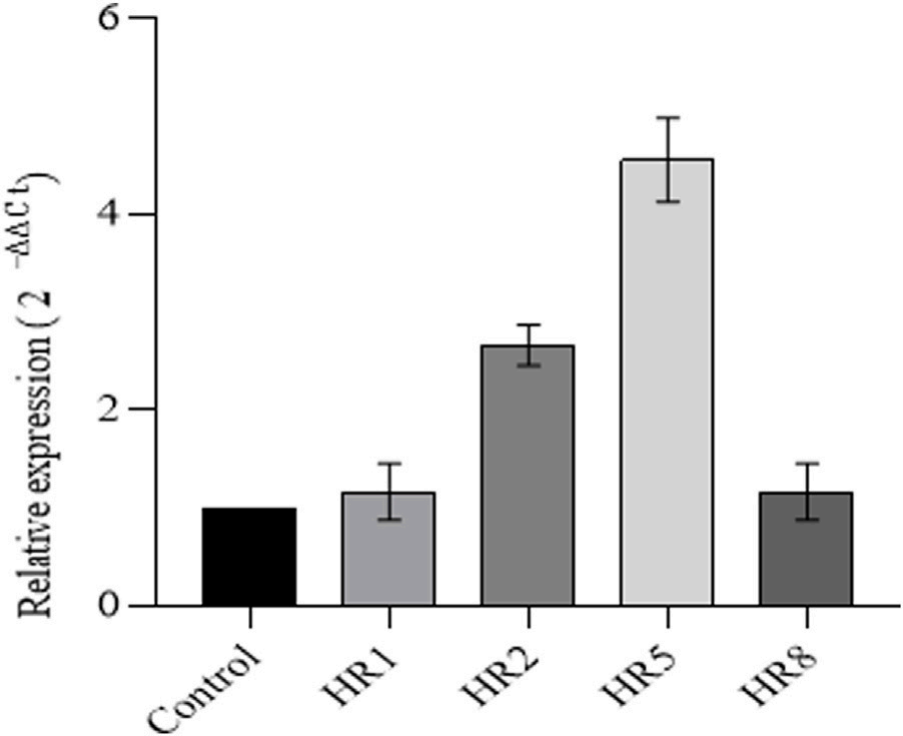
Real-time analysis of the VpVAN gene .Perform RT-qPCR analysis to determine the differential expression levels of VpVAN gene transcripts in transgenic hairy root lines compared to untransformed hairy root lines (control). The expression levels of the VpVAN gene in transgenic hairy root lines were consistent with the transcript levels of the internal control (Actin). The gene’s relative expression was determined using the 2-44ct method.

The mRNA extracted from the hairy roots of 60-day-old *B. vulgaris* was analyzed through qRT-PCR to confirm the expression of VpVAN. Different transgenic root lines showed varying levels of expression of VpVAN. The HR5 transgenic line exhibited the highest expression level, followed by the HR2 transgenic line (Figure 4). PCR was performed with actin gene amplification as a positive internal control. The HR5 transgenic line showed the maximum expression of VpVAN, which corresponded to the highest production of vanillin content.

### 3.6 Growth kinetics

**FIGURE 5:**
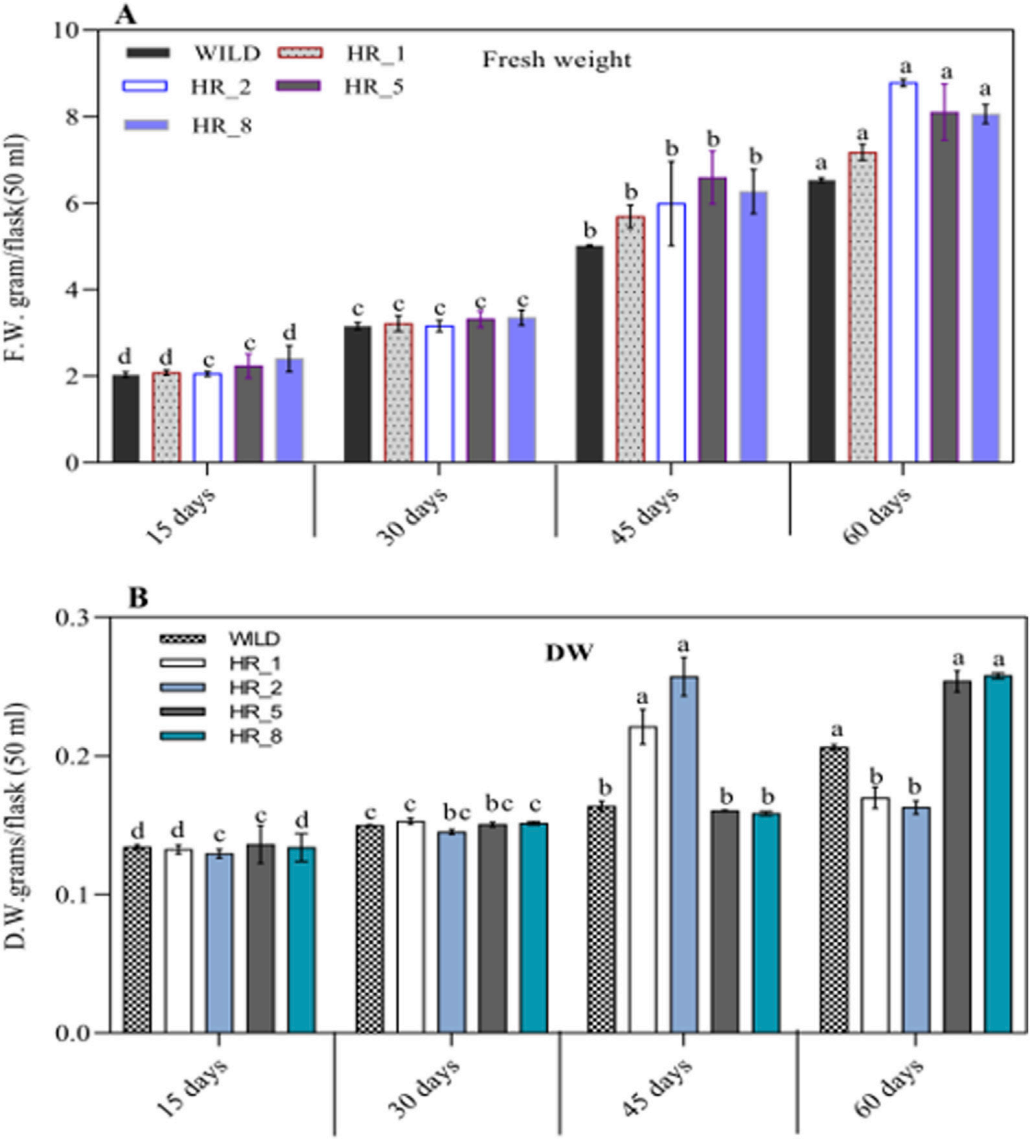
**(A, B)** The growth rates of four different lines of B. vulgaris hairy roots (HR_1, HR_2, HR_5, and HR_8) were measured over 60 days at 15, 30, 45, and 60 days. The results are reported as the mean ± standard deviation. The same letter in an individual experiment indicates no significant variations by Tukey’s test (p < 0.05).

After confirming the existence of hairy root lines at the molecular level, their growth was observed based on fresh and dry weights. The results showed (Figures 5A, B) that these roots absorbed nutrients from the medium and progressed to high branching after a 15-day-old. The exponential growth phase started from day 15 and continued until day 60, followed by a stationary phase lasting 15 days (up to day 75, data not shown) (Figures 2A–E). However, after day 75, the hairy roots entered the death phase (Figure 2F). This decline in later stages of growth and biomass was due to reduced nutrient availability, hairy root maturation, and excessive biosynthesis or release of metabolites into the medium, causing the roots to turn black at this stage. The growth of various root lines, including HR1, HR2, HR5, and HR8, exhibited significant differences when cultured in 50 mL of MS liquid medium. After 8 weeks (60 days) of growth, the root biomass ranged from 2.03 ± 0.061 to 8.78 ± 0.087 g/50 mL FW and 0.129 ± 0.003 to 0.258 ± 0.002 g/50 mL DW (Figures 5A, B). Out of the four root lines studied, the HR2 line displayed the highest growth and biomass, evident from the continuous increase in its hairy root growth/biomass (Figure 2E). The maximum biomass, i.e., 8.78 ± 0.087 g/50 mL FW and 0.258 ± 0.002 g/50 mL DW, was obtained after 8 weeks of culture, resulting in a ∼9.0-fold increase in the HR2 line over the initial inoculum, which was 2.2-fold higher than the control. Similar growth has been documented in Swertia chirayita (Mahendran et al., 2022) and Duboisia leichhardtii (Singh et al., 2018) earlier findings have indicated that the diversity seen among different hairy root clones is likely a result of variations in the expression levels of T-DNA genes integrated at various sites within the genome of the transformed hairy roots (Huang et al., 2016; Ghimire et al., 2019). The prevalence of position effect-mediated variations is high in transgenic plants (Sidorenko et al., 2003; Storb et al., 2022).

### 3.7 Levels of phenolic compounds detected in the hairy roots of *Beta vulgaris*

**FIGURE 6:**
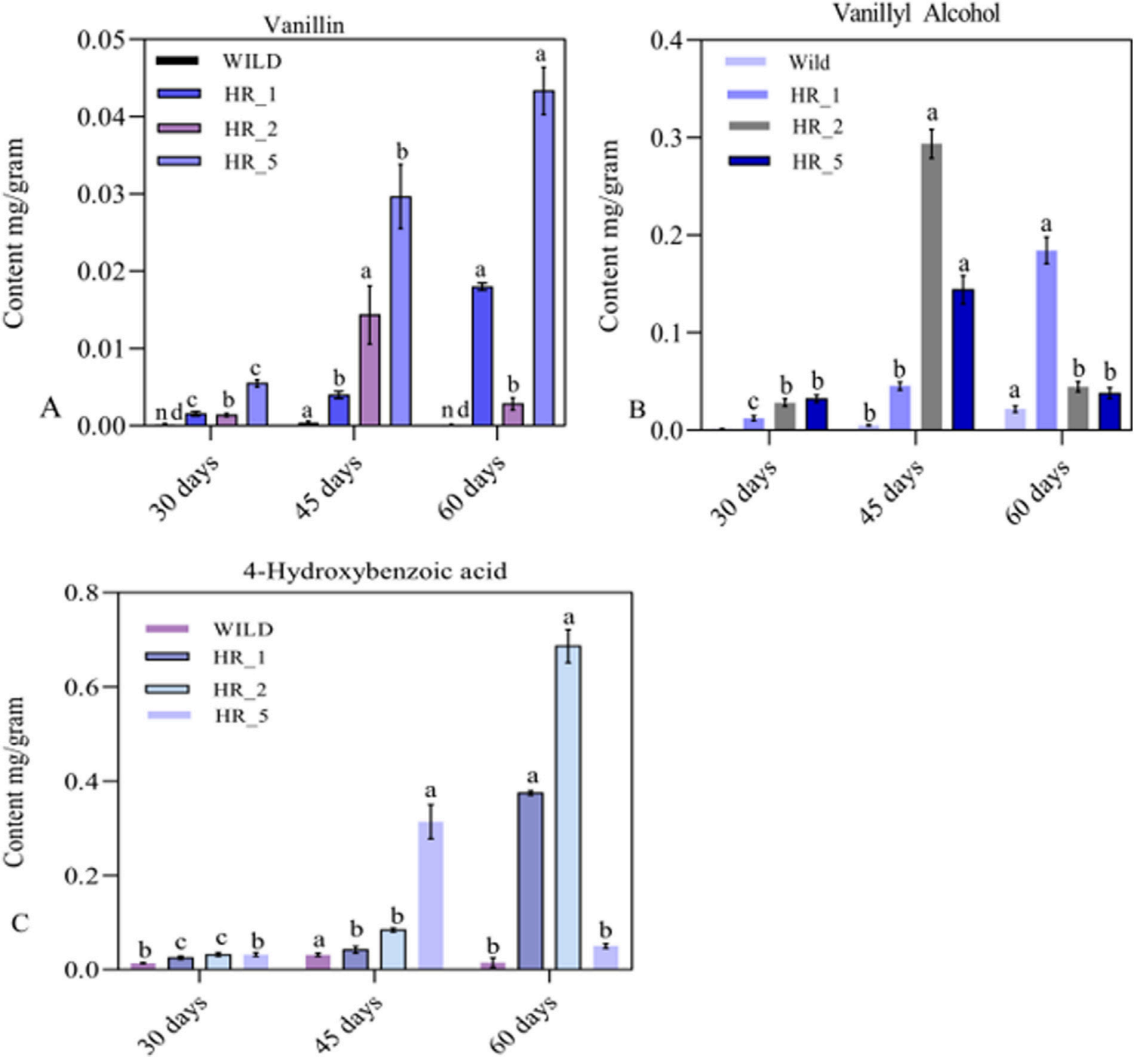
Variation of **(A)** vanillin, **(B)** vanillyl alcohol, and **(C)** 4-hydroxybenzoic acid content at different harvesting time intervals in the roots of **(A–C)** 3 positive and one negative (wild) lines of *Beta vulgaris*.

High-performance liquid chromatography (HPLC) was used to analyze three target phenolic compounds: Vanillin, Vanillyl alcohol, and 4-hydroxybenzoic acid. Vanillin, vanillyl alcohol, and 4-hydroxybenzoic acid (Sigma–Aldrich, United States) were co-injected with respective standards to identify peaks detected at 280 nm. Out of four transformed hairy root lines, only three (HR1, HR2, and HR5) produced vanillin and vanillin-related phenolic compounds. The concentration of vanillin varied in the genetically modified hairy roots (HR1, HR2, and HR5). The transgenic hairy root line HR5 exhibited the highest vanillin content, averaging 0.0430 (±0.003) mg/g dry weight (Figure 6A), while no vanillin was detected in the control group. The HR1 and HR2 root lines produced similar amounts of vanillin during the same period. However, their vanillin content was lower than the HR5 line nevertheless, all three transgenic lines accumulated significantly higher vanillin levels than the control. (Figure 6A). These results support earlier reports that vanillin production averaged 573.39 μg/g tissues higher than untransformed calli; the increase in vanillin levels was 190 times due to the *VpVAN* enzyme catalyzing the bioconversion of endogenous ferulic acid to vanillin (Chee et al., 2017). The calli of rice metabolically engineered with the VpVAN gene produce a vanillin content of 544.72 (±102.50) μg/g; in contrast, the vanillin content of wild-type rice calli averages 21.11 (±6.33) μg/g. (Arya et al., 2022). This result indicates that the *VpVAN* gene has facilitated vanillin from ferulic acid. Beetroot (*Beta vulgaris*) has been found to possess a considerable quantity of ferulic acid (FA) that is bound to the cell wall, comprising approximately 0.5%–1% of its dry weight (Clifford, 1999; Jiang et al., 2021); this suggests that bound ferulic acid and free ferulic acid may serve as a substrate for the VpVAN enzyme, which could catalyze vanillin production and related phenolic compounds in B. vulgaris. Our observations have revealed that the *VpVAN* gene played a crucial role in regulating phenolic compound production in transformed roots. Interestingly, the insertion of the VpVAN gene and considering the position effect of the gene increases the Vanillyl alcohol and 4-hydroxybenzoic acid in transgenic beetroot compared to nontransformed roots. The levels of 4-hydroxybenzoic acid and vanillyl alcohol in the HR2 line were significantly higher compared to the wild type. Specifically, the HR2 line exhibited 0.6867 ± 0.0351 mg/g of 4-hydroxybenzoic acid and 0.2933 ± 0.0153 mg/g of vanillyl alcohol (Figure 6B), while the wild type showed only 0.0310 ± 0.036 mg/g and 0.0207 ± 0.0030 mg/g, respectively (Figure 6C). Similarly, the *VpVAN* gene is expressed transiently in tobacco and stably in barley, producing vanillyl alcohol from ferulic acid (Gallage et al., 2014). The phenylpropanoid pathway produces free ferulic acid as an intermediate, which can be converted into various phenolic derivatives, including vanillic acid, vanillyl-CoA, and vanillin (Arya et al., 2022). These conversions occur through different possible routes for the bioconversion of ferulic acid to vanillin (Gallage and Møller, 2015). The vanillin synthase enzyme’s (VpVAN) reaction mechanism was similar to the HCHL enzyme from P. fluorescens. The reaction occurred in two stages. First, there was a hydration addition reaction, and a retro-aldol elimination reaction followed this (Paul et al., 2021; Jin et al., 2022). The expression of the HCHL gene in the hairy roots of N. tabacum and D. stramonium resulted in the production of novel compounds that were not present in the control plants, new products include glucoside and glucose ester of 4-hydroxybenzoic acid, vanillic acid glucoside, and glucoside of 4-hydroxybenzyl alcohol (Mayer et al., 2001; Mitra et al., 2002). The *B. vulgaris* plant exhibits the HCHL gene expression, thereby resulting in the synthesis of various compounds such as p-hydroxybenzoic acid (pHBA),p-hydroxybenzaldehyde (pHBAld),p-hydroxybenzoic acid glucose ester (pHBAGE), and p-hydroxybenzoic acid β-D-glucoside (pHBAG), (Rahman et al., 2009; Park et al., 2010). To explore novel ways of producing vanillin, a non-traditional method was employed, which utilized the HCHL gene to transform feruloyl-CoA, a precursor of lignin, into vanillin; the Vanillin two-step process; this approach was applied to hairy root cultures of *B. vulgaris* (Gasson et al., 1998; Singh et al., 2015).

### 3.8 Effect of MJ, SA, on the accumulation of increasing vanillin, vanillyl alcohol, and *4-*hydroxybenzoic acid

**FIGURE 7:**
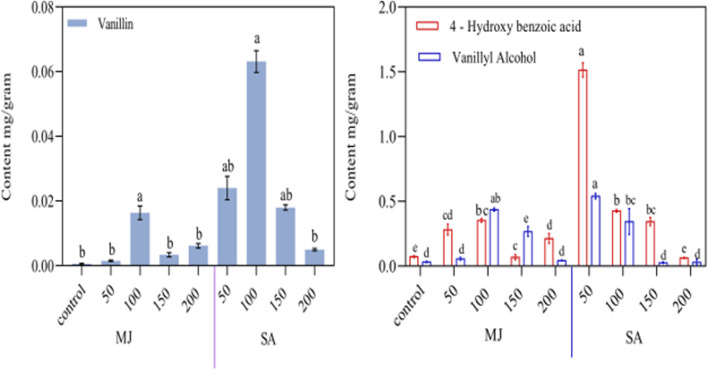
Effect of MJ, and SA on production in vanillyl alcohol, 4-hydroxybenzoic acid, vanillin, content after elicitation after 48 h *Beta vulgaris* hairy roots. Values are presented as mean ± standard deviation. The same letter in an individual experiment indicates no significant variations by Tukey’s test (p < 0.05).

This study investigated how vanillin, vanillyl alcohol, and 4-hydroxybenzoic acid levels in hairy root cultures responded to elicitation by MJ and SA. After treating HR2 line cultures with these elicitors, the HPLC method analyzed the metabolite contents after 48 h. The chromatograms showing the high concentrations of the three metabolites (4-hydroxybenzoic, vanillyl alcohol, and vanillin) are provided in the Supplementary File S2. Plants are being encouraged to produce more secondary metabolites by using elicitors. These are molecules that activate stress-regulating genes in order to stimulate the plant’s defense mechanisms. This trend has been observed in several recent studies (Bhaskar et al., 2021), demonstrated the effectiveness of elicitors, such as methyl jasmonate and salicylic acid, increasing the biosynthesis of metabolites, ((Xiao et al., 2009; Hao et al., 2015; Krishnan and Siril, 2018; Singh and Dwivedi, 2018; Shuang and Hong, 2020; Li et al., 2020; Naeini et al., 2021). In recent studies, elicitor treatments have increased root development and enhanced biomass in various plants, such as *Aster scaber*, *Centella asiatica*, and Salvia przewalskii (Ghimire et al., 2019; Baek et al., 2020; Li et al., 2020). In present research found that the accumulation of vanillyl alcohol and 4-hydroxy benzoic acid increased continuously but in the case of vanillin content, no significant difference lacking elicitor when hairy root culture was supplemented with MJ when hairy root culture was supplemented with MJ (50, 100, 150, and 200 μM) (Figure 7). The highest amount of 4-hydroxy benzoic acid (0.354 ± 0.15 mg/g DW) and vanillyl alcohol (0.437 ± 0.013 mg/g DW) was observed after applying MJ at 100 μM for 48 h, these values were around 5 and 13 times greater than the control (0.074 ± 0.007 and 0.033 ± 0.004 mg/g DW). Similarly, plants treated with MJ at 100 μM showed a rise of up to 177% in saponin content (Ghanati and Bakhtiarian, 2014). Moreover, swerchirin and 1, 2, 5, 6-tetrahydroxyxanthone content increased by 1.8 and 5.0 times, respectively (Mahendran et al., 2022). The hairy root culture of Salvia *miltiorrhiza* and Salvia castanea, F. tomentosa stib showed significant growth in tanshinones accumulation and gene expression of HMGS and HMGR upon treatment with MJ at 200 μM (Hou et al., 2021). MJ at 50 μM increased Rosmarinic acid accumulation by almost 1.7 times in Solenostemon scutellarioides (Sahu et al., 2022). In Salvia verticillata, RA content increased by more than 2.7-fold with 100 μM MJ exposure (Rahmani and Radjabin, 2023). According to Baek et al., 2020 found that applying MJ at a concentration of 400 μM to *C. asiatica* hairy root cultures resulted in a significant increase in the content of triterpenoids, particularly madecassoside.

Salicylic acid has been used on various plants to synthesize specific secondary metabolites (Ali, 2021). SA has been utilized to stimulate the production of secondary metabolites and increase the capsaicinoid content *in vitro* cultures of the *Capsicum* genus (Sudha and Ravishankar, 2002). The production of capsaicinoids can arise from the phenylpropanoid pathway and related compounds, which may impact the plant’s defense mechanisms (Hao et al., 2016). Activation of PAL by phospholipid signaling influences SA-induced vanillin production in cell suspension of Capsicum chinense (Rodas-Junco et al., 2013) results indicate that SA activates PAL activity, resulting in vanillin synthesis. In our study, we investigated the effects of different concentrations of SA (50, 100, 150, and 200 μM) on hairy roots and their ability to produce certain compounds. We observed a dose-dependent increase in the content of 3 compounds: vanillin, vanillyl alcohol, and 4-hydroxybenzoic acid, after 48 h of SA treatment (Fig. 5A & B). Our research has shown that the highest levels of vanillin (0.063 mg/g) and vanillyl alcohol (0.540 ± 0.21), 4-hydroxybenzoic acid (1.515 ± 0.055) were detected at 50 μM salicylic acid (SA) concentrations, respectively, after 48 h of treatment. These findings indicate a 1.5-fold increase in vanillin, a 1.8-fold increase in vanillyl alcohol, and a 2.2-fold increase in 4-hydroxybenzoic acid compared to untreated elicitor transgenic root. The similarly of SA at different concentrations has been found to promote the biosynthesis of vanillin, ferulic acid, and caffeic acid, leading to a considerable rise in their production compared to the control (Vyas and Mukhopadhyay, 2018). The study’s objective was to explore how vanillin, vanillyl alcohol, and 4-hydroxybenzoic acid levels in leaf-derived hairy root cultures would be treated by elicitors, such as MJ and SA. After fortifying the cultures with elicitors, we analyzed the metabolite contents 48 h later. There has been a recent emphasis on increasing secondary metabolite biosynthesis in plants using elicitors, which are molecules that activate stress-regulating genes to trigger plant defense mechanisms. This trend has been observed in several recent studies. Previously, treating safflower with 250 μM salicylic acid enhanced secondary metabolites such as flavonoids, anthocyanin, phenol, and phenylalanine ammonia-lyase activity (Chavoushi et al., 2020). Papaver armeniacum showed the highest contents of papaverine and noscapine at 100 μM SA (Naeini et al., 2021). The study observed a significant increase in the accumulation of vanillin, vanillyl alcohol, and 4-hydroxybenzoic acid content. The levels were enhanced up to 215-fold, 13-fold, and 21-fold, respectively, compared to non-transgenic hairy roots.

## 4 Conclusion

*Beta vulgaris* hairy root culture was established using *A. rhizogenes* (LBA 9402 strain). The study investigated the effects of *A. rhizogenes* cell density, infection time, and acetosyringone concentrations and found that an optimum root induction efficiency of 64.33% ± 4.041% was observed at 0.8 (OD_600_) *A. rhizogenes* cell density +12 min infection time with 75 µM of acetosyringone. The study successfully achieved the heterologous transfer of the VpVAN gene, which produced vanillin, vanillyl alcohol, and 4-hydroxybenzoic acid in the hairy roots of *B. vulgaris*. Elicitation with methyl jasmonate and salicylic acid enhanced the yield of these compounds. This alternative method helped address the increasing demand for these compounds and reduced the cost of natural vanillin production.

## Data Availability

The datasets presented in this study can be found in online repositories. The names of the repository/repositories and accession number(s) can be found in the article/Supplementary Material.
