# Supplementary material for: Metabolic engineering of hairy root cultures in Beta vulgaris for enhanced production of vanillin, 4-hydroxybenzoic acid, and vanillyl alcohol
Source: Front Bioeng Biotechnol. 2024 Oct 2;12:1435190. doi: 10.3389/fbioe.2024.1435190 (PMC11480924; doi:10.3389/fbioe.2024.1435190)
Supplement: Supplementary file 1 [file Table1.DOCX]

**Table S1**

**Oligonucleotide sequences used for the semi-quantitative RT-PCR in this study**

| Direction Primer sequence (5′- 3′) |
| --- |

**Primers used for semi-quantitative-PCR**

**Molecular analysis of the hairy roots of B. vulgaris.**

**For rolC**

Forward ATGGATCCCAAATTGCTATTCC 3

Reverse GAGAGTCGCAGGGTTAGGTCTG

**For VpVAN**

Forward ATGGCAGCTAAGCTCCTCTTCT

Reverse CTACACAGCCACAATGGGATA

**Primers used for quantitative RT-PCR**

**For VpVAN**

Forward TCTTGCCGTCGGTTATGGA

Reverse CCCCAATTTGTACCCCATGA

**For Actin**

Forward ACCTTCCAGCAGATGTGGAT′

Reverse AGCACTTACGGTGGACAATTA
