# Supplementary material for: Metabolic engineering of hairy root cultures in Beta vulgaris for enhanced production of vanillin, 4-hydroxybenzoic acid, and vanillyl alcohol
Source: Front Bioeng Biotechnol. 2024 Oct 2;12:1435190. doi: 10.3389/fbioe.2024.1435190 (PMC11480924; doi:10.3389/fbioe.2024.1435190)
Supplement: Supplementary file 2 [file DataSheet1.docx]

**Supplementary file (S2) of HPLC chromatogram**


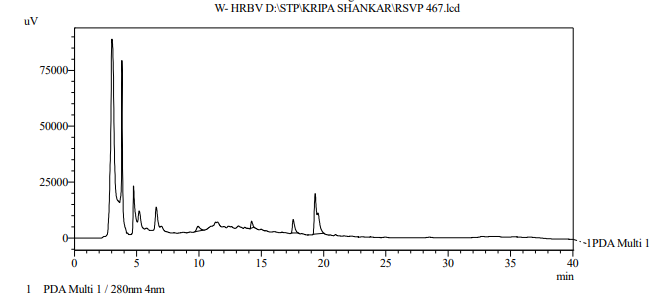


**Wild**

4HBA

VA


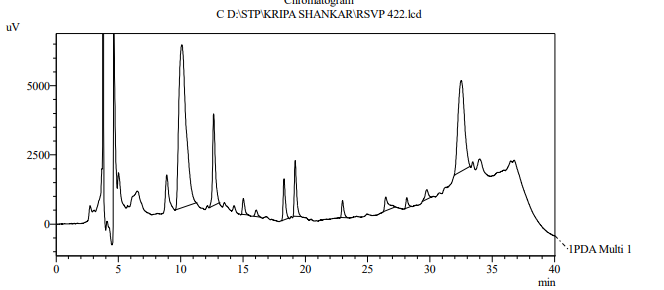


4HBA

VA

Vanillin

**50 μM SA**


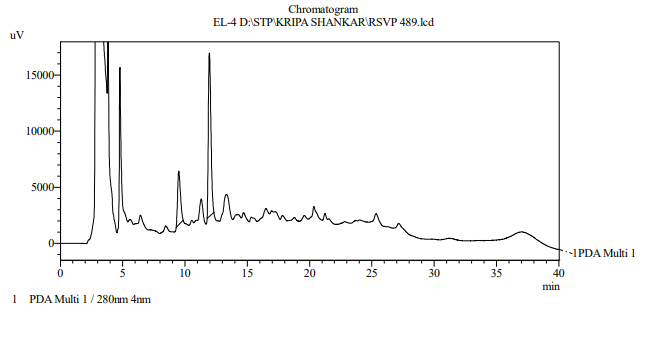


**100 μM MJ**

4HBA

VA

Vanillin


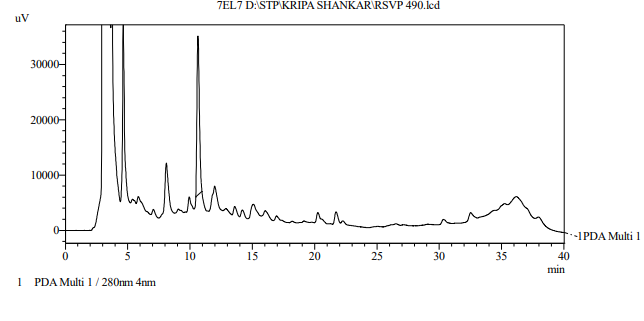


4HBA

VA

Vanillin

**HR2**

Not detected


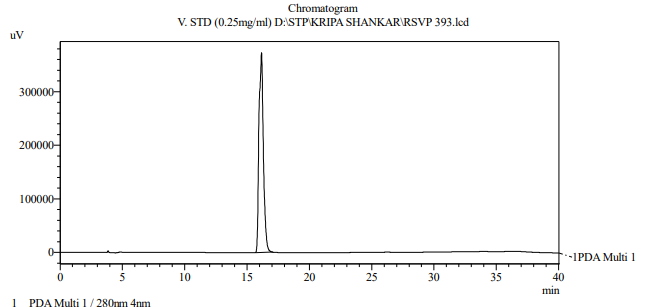


Vanillin


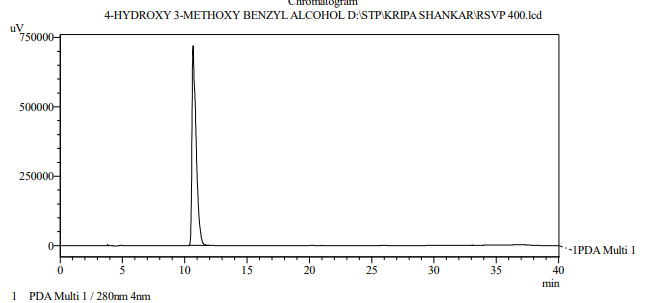

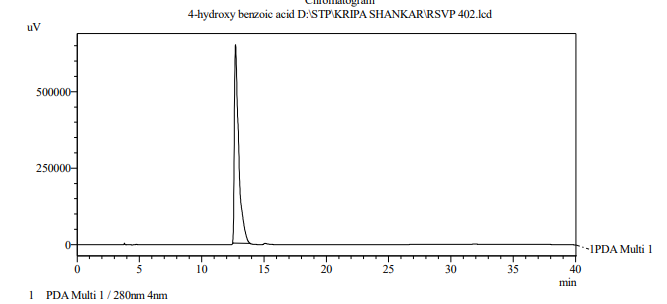


4-hydroxy benzoic acid

Vanillyl Alcohol

**e**

**f**

**g**

**a**

**b**

**c**

**d**

**Fig.S2.** HPLC chromatogram of the high content of metabolites, including 4-hydroxybenzoic acid, vanillyl alcohol, and vanillin, was found in putative transgenic HR2 lines treated with 100 μM methyl jasmonate (MJ) (a) and 50 μM salicylic acid (SA) (b). At the same time, untreated transgenic roots (c) and non-transgenic root extracts (d) were also analyzed for comparison. HPLC chromatogram of the standard. (e) Vanillyl Alcohol (f) 4-hydroxybenzoic acid. (g) Vanillin.
